# Unlocking the Long-Term Effectiveness of Benralizumab in Severe Eosinophilic Asthma: A Three-Year Real-Life Study

**DOI:** 10.3390/jcm13103013

**Published:** 2024-05-20

**Authors:** Laura Pini, Diego Bagnasco, Bianca Beghè, Fulvio Braido, Paolo Cameli, Marco Caminati, Cristiano Caruso, Claudia Crimi, Gabriella Guarnieri, Manuela Latorre, Francesco Menzella, Claudio Micheletto, Andrea Vianello, Dina Visca, Benedetta Bondi, Yehia El Masri, Jordan Giordani, Andrea Mastrototaro, Matteo Maule, Alessandro Pini, Stefano Piras, Martina Zappa, Gianenrico Senna, Antonio Spanevello, Pierluigi Paggiaro, Francesco Blasi, Giorgio Walter Canonica, on behalf of the SANI Study Group

**Affiliations:** 1 ASST Spedali Civili of Brescia, 25123 Brescia, Italy; 2 Department of Clinical and Experimental Sciences, University of Brescia, 25122 Brescia, Italy; 3 Allergy and Respiratory Diseases Clinic, IRCCS Policlinico San Martino, 16132 Genova, Italy; 4 Department of Medical and Surgical Sciences, Maternal, Infant and Adult, University of Modena and Reggio Emilia, 41124 Modena, Italy; 5 Respiratory Diseases Unit, Department of Medical Sciences, Azienda Ospedaliera-Universitaria Senese, 53100 Siena, Italy; 6 Department of Medicine, University of Verona, 37134 Verona, Italy; 7 Asthma Center and Allergy Unit, Verona University Hospital, 37126 Verona, Italy; 8 Allergologic Unit, Policlinico Agostino Gemelli, 00168 Rome, Italy; 9 Respiratory Medicine Unit, Policlinico “G. Rodolico-San Marco” University Hospital, 95123 Catania, Italy; 10 Department of Cardiac, Thoracic and Vascular Sciences, University of Padova, 35122 Padova, Italy; 11 Pneumologic Unit, Department of Medical Specialties, Nuovo Ospedale delle Apuane, 54100 Massa, Italy; 12 Pneumologic Unit, Ospedale di Montebelluna, 31044 Montebelluna, Italy; 13 Pneumologic Unit, Ospedale Borgo Trento, 37126 Verona, Italy; 14 Department of Medicine and Surgery, Respiratory Diseases, University of Insubria, 21100 Varese, Italy; 15 Department of Cardio-Respiratory Medicine and Rehabilitation, Division of Pulmonary Rehabilitation, Istituti Clinici Scientifici Maugeri, IRCCS, 21049 Tradate, Italy; 16 Department of Emergency, Anaesthesiological and Resuscitation Sciences, University Cattolica Sacro Cuore, 29122 Rome, Italy; 17 Department of Surgery, Medicine, Molecular Biology and Critical Care, University of Pisa, 56124 Pisa, Italy; 18 Department of Pathophysiology and Transplantation, University of Milano, 20122 Milan, Italy; 19 Respiratory Unit and Cystic Fibrosis Center, Fondazione IRCCS Cà Granda Ospedale Maggiore Policlinico di Milano, 20122 Milan, Italy; 20 Personalized Medicine Center, Asthma and Allergology, Humanitas Research Hospital, 20089 Rozzano, Italy; 21 Department of Biomedical Sciences, Humanitas University, 20090 Pieve Emanuele, Italy

**Keywords:** severe eosinophilic asthma, benralizumab, effectiveness, long term, clinical remission

## Abstract

**Background:** Benralizumab has been shown to restore good control of severe eosinophilic asthma (SEA). Robust data on benralizumab effectiveness over periods longer than 2 years are scarce. **Methods:** This retrospective multicentric study was conducted on 108 Italian SEA patients treated with benralizumab for up to 36 months. Partial and complete clinical remission (CR) were assessed. Data were analyzed with descriptive statistics or using linear, logistic, and negative binomial mixed-effect regression models. **Results:** At 36 months, benralizumab reduced the exacerbation rate by 89% and increased the forced expiratory volume in 1 second (FEV_1_) (+440 mL at 36 months, *p* < 0.0001). Benralizumab improved asthma control as well as sinonasal symptoms in patients with chronic rhinosinusitis with nasal polyposis (CRSwNP). Up to 93.33% of patients either reduced or discontinued OCS; benralizumab also decreased ICS use and other asthma medications. Overall, 84.31% of patients achieved partial or complete CR. **Conclusions:** Benralizumab improved asthma and sinonasal outcomes up to 36 months. These findings support the potential of benralizumab to induce CR, emphasizing its role as a disease-modifying anti-asthmatic drug for the management of SEA. Further research is warranted to expand these findings by minimizing data loss and assessing benralizumab’s long-term safety.

## 1. Introduction

Severe asthma (SA) is a debilitating chronic disorder that affects approximately 10% of asthma patients worldwide [1,2,3]. Due to its heterogeneous nature, distinct phenotypes and endotypes have been identified and prompted the sub-classification of the disease according to clinical characteristics and functional and inflammatory parameters [4]. Severe eosinophilic asthma (SEA) is one of the predominant subtypes of SA [5,6]; its pathophysiology is defined by an extensive type 2 (T2) inflammatory process mainly driven by the proliferation and activation of eosinophils. Accordingly, eosinophil number is increased in blood and sputum of SEA patients; other key characteristics are a scarce respiratory function that further deteriorates over time, and recurrent and/or life-threatening exacerbations [7,8,9]. Given the high burden of its manifestations and the poor prognosis, SEA has a devastating impact on patients’ quality of life (QoL), which can be further aggravated by the presence of comorbidities, among which chronic rhinosinusitis with nasal polyposis (CRSwNP) is one of the most frequently observed [10,11].

The recommended background therapies, which include inhaled corticosteroids (ICS) and a second controller (usually a long-acting beta2-agonist [LABA]) [12] are not always effective in managing SEA symptoms. Based on their potent anti-inflammatory action, oral corticosteroids (OCS) have been traditionally added to background medications in cases of inadequate asthma control, to prevent exacerbations. However, given the cumulative risk of significant adverse effects and mortality associated with their usage, even moderate dosages of OCS should be avoided [12,13,14,15,16].

The development of several biological therapies has represented a giant step forward in the treatment of T2-high SA. Six biologics have thus far received approval (omalizumab, mepolizumab, reslizumab, benralizumab, dupilumab, tezepelumab); by targeting distinct pathways involved in the pathophysiology of the disease, they ensure superior efficacy and safety than OCS [17]. Based on their different mechanism of action (MoA), each one of these pharmacological agents is expected to be more successful in patients whose asthma is predominantly sustained by the corresponding inflammatory endotype. However, SA patients frequently show overlapping T2-high features [18]; as a result, precise pheno-endotypization is required to identify the driving pathway of the disease and anticipate the most effective biologic treatment.

Overall, the great clinical outcomes displayed by SA patients treated with biologic therapies have highlighted the potential of reaching a status of remission from the disease. To clarify this concept, a consensus on the criteria that define clinical remission (CR), both complete (cCR) and partial (pCR), was recently reached by a panel of experts from the Severe Asthma Network Italy (SANI) study group, allowing for the standardized assessment of patients regardless of the biological treatment received [19,20].

Benralizumab is a monoclonal antibody (mAb) approved for the treatment of SEA [21]. It is a humanized afucosilated immunoglobulin (Ig) Gk1 mAb that binds both interleukin 5 receptor alpha (IL-5Rα) and Fc gamma receptor IIIa (FcγRIIIa), expressed abundantly by eosinophils and natural killer (NK) cells, respectively. The simultaneous recognition of the two receptors allows benralizumab to activate antibody-dependent cell-mediated cytotoxicity (ADCC), a process through which NK cells induce the apoptosis of eosinophils [22,23]. The consequent nearly complete depletion of eosinophils differentiates benralizumab from the anti-IL-5 mAbs mepolizumab and reslizumab [24], and determines the well-established efficacy of benralizumab in SEA patients [25]. In a recent study, benralizumab has been shown to have profound immunological effects that are not limited to eosinophil apoptosis but include an increase in NK cell proliferation, maturation and cytotoxic activity, and the modulation of T cell subsets. Intriguingly, the number of circulating CD3 + T cells and activated NK cells significantly correlated with improvement in lung function parameters in benralizumab-treated SEA patients [26]. These results deepen our understanding of benralizumab MoA, which appears to be more complex than what was traditionally thought. These data suggest that the improvement in respiratory outcomes mediated by benralizumab may be due to a profound immunological modulation that takes place even in the absence of eosinophils.

To date, the 5-year-long MELTEMI trial represents the longest study evaluating the effects of benralizumab on SEA patients; the results indicated that benralizumab was safe and effective in eliminating exacerbations in up to 59% of patients during the entire extension period (BORA and MELTEMI studies, up to 4 years of treatment) [27]. Few real-life studies have investigated the benralizumab effectiveness over 20-month–4-year-long periods [25,28,29,30,31,32,33]. A marked and long-lasting reduction in exacerbations has been consistently shown across all studies, with impressive results obtained from the Italian ANANKE study, showing reductions of 94.9% and 96.9% in all and severe annual exacerbation rate (AER), respectively, after 96 weeks of treatment [25]. Positive outcomes have also been demonstrated in lung function and OCS reduction; however, the extent and the durability of benralizumab effectiveness on respiratory outcomes for periods longer than 2 years is still uncertain [31,33]. Recently published data from the phase IV randomized clinical trial (RCT) SHAMAL revealed that the benralizumab treatment was associated with a substantial reduction in the dose of background ICS, showcasing an additional clinical benefit of the anti-IL-5R therapy [34]. This Italian multicentric retrospective study includes a cohort of 108 SEA patients treated with benralizumab for up to 3 years. The data show changes in multiple clinical outcomes and provide a comprehensive analysis, as well as novel evidence, of the long-term effectiveness of benralizumab.

## 2. Materials and Methods

This is an observational retrospective study; data were collected from 9 Italian SANI centers specialized in the treatment of SA (Brescia, Catania, Modena, Montebelluna, Padova, Varese, Verona, Siena, Rome, Italy).

SA was diagnosed according to the European Respiratory Society (ERS) and the American Thoracic Society (ATS) guidelines [1]. Benralizumab was prescribed to adult patients as per Italian clinical practice, according to the eligibility and reimbursement criteria dictated by the therapeutic plan and previously established by the Italian regulatory drug agency (Agenzia Italiana del Farmaco, AIFA, Rome, Italy). To be eligible for benralizumab treatment, patients must have had any blood eosinophil count (BEC) ≥300 cells/µL in the absence of OCS treatment; such a BEC value may have been measured at any time before benralizumab initiation. In addition to this criterion, patients must meet one of the two following conditions: (1) at least two exacerbations in the previous 12 months despite maximum dose of inhaled therapy (steps 4–5 of the GINA document) and treated with systemic steroid or requiring hospitalization; (2) continuous OCS treatment received during the previous year in addition to maximal inhaled therapy [35]. Benralizumab was given subcutaneously at a dose of 30 mg; after the first three doses, which were administered every four weeks, benralizumab was administered every eight weeks.

A total of 108 patients were enrolled between January 2018 and February 2021; follow-up visits took place at 6, 12, 24, and 36 months after benralizumab initiation. Personal information including socio-demographic, clinical, functional and laboratory data were recorded as per clinical practice immediately before benralizumab initiation (i.e., at baseline) and during follow-up visits. Data were retrospectively collected from medical charts between April and June 2023. More specifically, the baseline characteristics included age, gender, body mass index (BMI), smoking habit, age at asthma diagnosis, age at benralizumab initiation, comorbidities, use and dose of asthma therapies (background inhaled medications, OCS, biologics received prior to benralizumab use), laboratory tests (BEC, IgE, fractioned exhaled nitric oxide [FeNO]), exacerbations (expressed as AER), as determined by treating physicians, and pre- and post-bronchodilator (BD) lung function parameters including forced expiratory volume in 1 second (FEV_1_), and forced vital capacity (FVC). All respiratory measurements are pre-BD, unless otherwise specified.

Various patient reported outcomes (PROs) were also used to assess patients’ asthma control (asthma control test, ACT, asthma control questionnaire-6, ACQ), and QoL (asthma quality of life questionnaire, AQLQ). The severity of sinonasal symptoms was specifically investigated in comorbid patients with CRSwNP using visual analogue scale (VAS) and sinonasal outcome test 22 (SNOT-22). The absolute differences in AER between 0 and 12 months and between 0 and 36 months were computed. Changes in laboratory parameters, AER, lung function parameters, PROs, background therapies, and OCS were evaluated over time. The number and percentages of patients reaching either pCR or cCR (defined according to the criteria detailed by Canonica et al. [19]) were determined at 12, 24 and 36 months; CR was calculated for each time point either from the corresponding previous time point (i.e., from baseline to 12 months; from 12 months to 24 months; and from 24 months to 36 months) or from baseline. pCR was defined by three criteria: (1) no use of OCS, accompanied by two of the following: either (1) good asthma control defined by ACT score ≥ 20; and/or (2) elimination of exacerbations; and/or (3) pulmonary stability. cCR was achieved by patients who met all four of the following criteria: (1) no use of OCS; (2) good asthma control defined by ACT score ≥ 20; (3) elimination of exacerbations; and (4) pulmonary stability [19].

Informed consent was obtained from all patients involved in the study. This was a SANI study; the study was conducted in conformity with the Declaration of Helsinki and was approved by the Central Ethics Committee for the SANI Network.

### Statistical Analyses

For descriptive analyses, continuous variables were given as the mean with standard deviations (SD), or a median with range or interquartile range (IQR) and categorical variables were expressed as the number of subjects (n) and percentage values.

Possible over-dispersion for count values was assayed using a formal test based on the code from Gelman and Hill (2007) [36]. Linear, logistic, and Poisson mixed-effect regression models were performed on the continuous, dichotomy, and count values, respectively, to evaluate changes in AER, lung function, PROs scores, OCS, and use of asthma medication over time. Regression coefficients, odds ratios (ORs), and exponential regression coefficients associated with each outcome were calculated with 95% confidence intervals (CI) for each factor.

The center and subject variability were considered to be random effects in all mixed-effect regression models. The likelihood ratio test was used as a test of statistical significance and *p*-values were adjusted for multiple comparisons using the Holm correction method.

Differences, with a *p*-value less than 0.05, were selected as significant. Data were acquired and analyzed in R version v4.3.1 software environment [37].

## 3. Results

### 3.1. Patients’ Characteristics at Baseline

The demographics, biochemical, and clinical characteristics of the study participants at baseline are summarized in Table 1. Briefly, a total of 108 patients (59.26% female, mean age 55.96 years) took part in this study; the population was highly comorbid, with 94 patients (87.04%) with at least one comorbidity. Chronic rhinosinusitis (CRS), and specifically CRSwNP, was the most common comorbidity, being present in 65 patients (60.19%). A total of 28 out of 104 patients (26.96%) had bronchiectasis and 4 out of 96 patients (4.71%) had eosinophilic granulomatosis with polyangiitis (EGPA). The mean age at asthma diagnosis was 35.68 years (15.95). All patients used ICS and LABA as background therapies, the mean ICS dose used by patients was high (1006.93 mcg/day (402.56)) and the majority of patients required additional asthma medications, including long-acting muscarinic antagonists (LAMA) (79 out of 107 patients, 73.83%), anti-leukotrienes (anti LT) (52 out of 107 patients, 48.60%), theophylline (6 out of 106 patients, 5.66%). OCS were taken by 67 patients (62.04%) at a mean dose of 7.95 mg/day (7.42); a total of 41 patients used the OCS dose higher than 5 mg/day (61.19%). Of note, benralizumab was the first biologic therapy used in 89 patients (82.41%), while 19 patients were switched to benralizumab after being treated with other biologics (either omalizumab or mepolizumab).

Biochemical analyses revealed that patients had a median BEC of 600 cells/mm^3^ (28–3350) (*n* = 104), a median total IgE level of 209 IU/mL (4.9–1940) (*n* = 91), and a mean FeNO of 61.95 ppb (55.66) (*n* = 55). The frequency of asthma exacerbations approached four events/year, with a mean AER of 3.84 (3.18) (*n* = 107); the phenotype of exacerbations was assessed in 45 patients, among which 14 (31.11%) had exacerbations of non-infectious nature, whereas the rest of patients experienced either viral (15.56%) or bacterial-induced (53.33%) exacerbations. Patients were also characterized by suboptimal lung function, showing a mean FEV_1_/FVC ratio of 54.11 (24.87) (*n* = 91), a mean FEV_1_ of 1.94 L (0.83) (*n* = 101), and a mean predicted FEV_1_ of 74.50% (20.68) (*n* = 86).

Overall, asthma was uncontrolled despite the use of multiple medications, and patients’ QoL was poor, as demonstrated by the low scores achieved in various PROs, including ACT (*n* = 104), ACQ (*n* = 39) and AQLQ (*n* = 31) (mean values of 14.41, 2.33 and 3.76, respectively). VAS (*n* = 60) and SNOT-22 (*n* = 57) were administered to comorbid patients with CRSwNP and showed a mean score of 5.14 (3.13) and 47.21 (20.67), respectively, indicative of moderate sinonasal symptom severity [38].

### 3.2. Benralizumab Reduced Exacerbations and Inflammatory Markers

**Figure 1:**
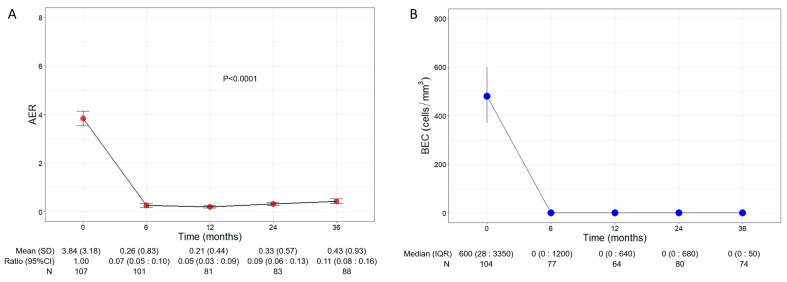
Change in AER (**A**) and BEC (**B**) during the treatment with benralizumab. Data were recorded at baseline and at 6, 12, 24, and 36 months. Mean (SD), *n* values and exponential beta regression coefficients (i.e., ratio) with 95% CI, or median (IQR) and *n* values are reported for each time point.

Patients experienced a significant and remarkable reduction in AER throughout the three years of treatment with benralizumab (*p* < 0.0001, Figure 1A); baseline AER declined from a mean of 3.84 (3.18) to 0.26 (0.83) (0.07, 95% CI 0.05:0.10) already after 6 months of treatment and remained low at all time points, reaching 0.43 (0.93) (0.11, 95% CI 0.08:0.16) at 36 months. Compared with the baseline, the decline in AER amounted to 93%, 95%, 91%, and 89% at 6, 12, 24, and 36 months, respectively.

Consistent with its anti-eosinophilic effect, benralizumab treatment induced an almost complete depletion of BEC that was sustained over time, with a median BEC of 0 (0:50) displayed at all time points (Figure 1B). The drop in BEC was accompanied by a persistent reduction in FeNO, which decreased from a mean of 61.95 ppb (55.66) at baseline to 42.27 ppb (32.31) at 36 months (*p* = 0.0001, Table S1).

### 3.3. Benralizumab Improved Lung Function

**Figure 2:**
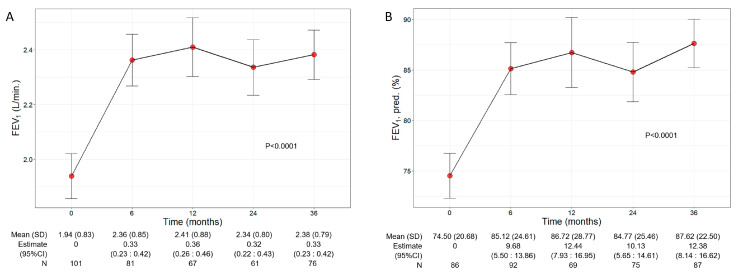
Change in FEV_1_ volume (**A**) and percentage of predicted (**B**) during the treatment with benralizumab. Data were recorded at baseline and at 6, 12, 24, and 36 months. Mean (SD), *n* values and regression coefficients (i.e., estimate) with 95% CI are reported for each time point.

Patients treated with benralizumab ameliorated their lung function throughout the 36-month period, with a significant increment observed in FEV_1_ (*p* < 0.0001) (Figure 2). The mean volume of FEV_1_ increased from 1.94 L (0.83) at baseline to 2.38 L (0.79) at 36 months (+440 mL), with the maximal value of 2.41 L (0.88) recorded at 12 months (Figure 2A), while percentage of predicted FEV_1_ peaked at 36 months (87.62%, with a mean increase of 13.12% from baseline) (12.38, 95% CI 8.14:16.62) (Figure 2B). Variations in other respiratory measurements are reported in Table S1.

### 3.4. Benralizumab Increased Asthma Control and QoL

**Figure 3:**
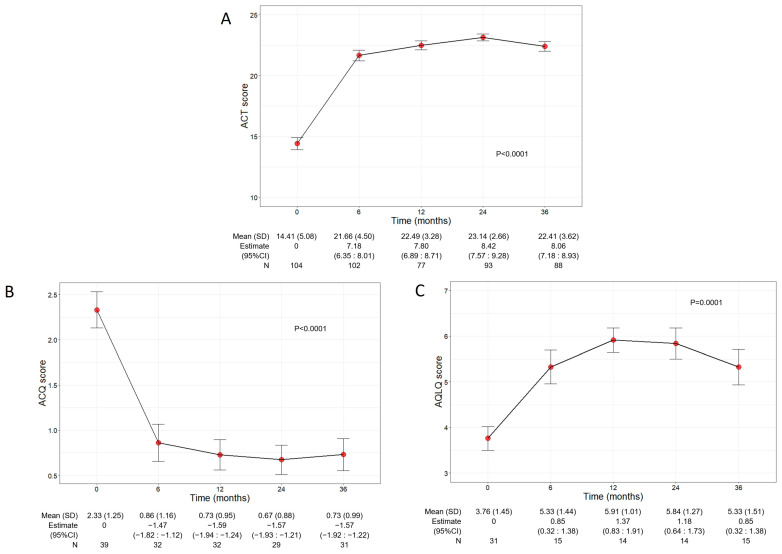
Change in ACT (**A**), ACQ (**B**), and AQLQ (**C**) scores during the treatment with benralizumab. Data were recorded at baseline and at 6, 12, 24, and 36 months. Mean (SD), *n* values and regression coefficients (i.e., estimate) with 95% CI are reported for each time point.

Asthma control and QoL significantly ameliorated during benralizumab treatment, with net improvements observed already at 6 months after the start of the treatment (Figure 3A). In detail, the mean ACT score was 14.41 (5.08) at baseline and increased to 21.66 points (4.50) at 6 months (7.18, 95% CI 6.35:8.01); the ACT score remained either stable or further increased throughout the treatment period, indicating a durable good control of asthma. In addition, benralizumab enhanced the patients’ QoL as determined by the significant changes of both ACQ and AQLQ scores over time (mean ACQ: from 2.33 to 0.73, *p* < 0.0001; mean AQLQ: from 3.76 to 5.33, *p* = 0.0001) (Figure 3B,C).

### 3.5. Benralizumab Alleviated Sinonasal Symptoms in Comorbid Patients

**Figure 4:**
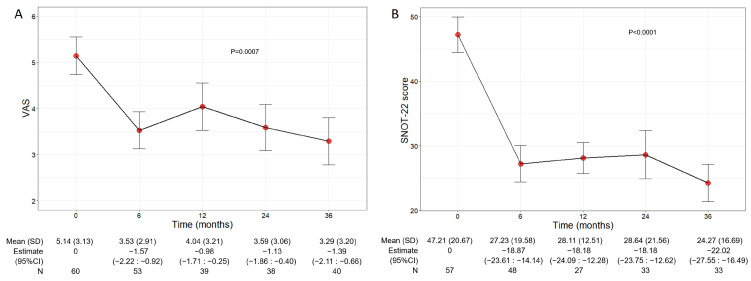
Change in VAS (**A**) and SNOT-22 (**B**) score during the treatment with benralizumab. Data were recorded at baseline and at 6, 12, 24, and 36 months. Mean values (SD), *n* and regression coefficients (i.e., estimate) with 95% CI are reported for each time point.

Beyond the asthma control improvement, benralizumab also decreased the severity of sinonasal symptoms in the subset of comorbid patients with CRSwNP. The mean VAS score significantly declined over the treatment period (*p* = 0.0007), decreasing from 5.14 (3.13) at baseline to 3.29 (3.20) at 36 months (−1.39, 95% CI −2.11:−0.66) (Figure 4A). In parallel, the mean SNOT-22 score also significantly dropped (*p* < 0.0001) from 47.21 (20.67) at baseline to 24.27 (16.69) at 36 months (−22.02, 95% CI −27.55:−16.49) (Figure 4B).

### 3.6. Benralizumab Decreased the Use of OCS

**Figure 5:**
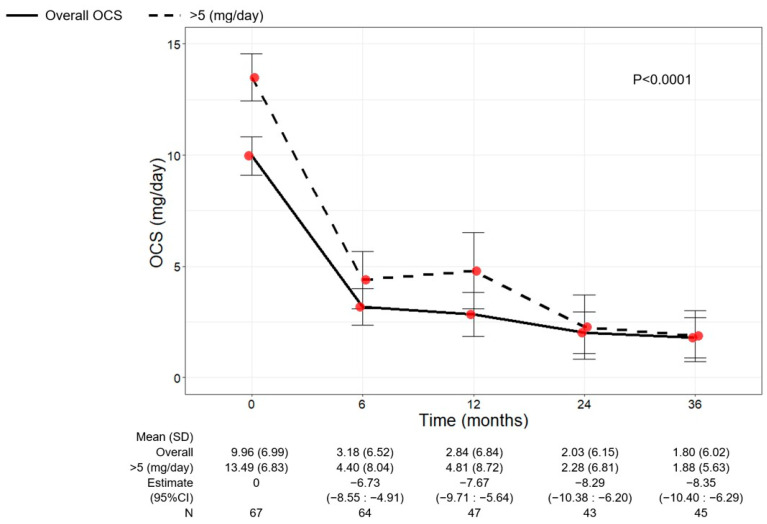
Change in OCS dose in the overall patient population and in patients with baseline OCS dose > 5 mg/day during the treatment with benralizumab. Data were recorded at baseline and at 6, 12, 24, and 36 months. Mean (SD), *n* values and regression coefficients (i.e., estimate) with 95% CI are reported for each time point.

As shown in Figure 5, a significant reduction in OCS use was observed over time (*p* < 0.0001). The mean dose of OCS decreased to 3.18 mg/day (6.52) at 6 months (−6.73, 95% CI −8.55:−4.91) and continued to decline, reaching a mean dose of 1.80 mg/day (6.02) at 36 months (−8.35, 95% CI −10.40:−6.29). A total of 39 out of 64 (60.94%) eliminated the use of OCS already after 6 months from the start of benralizumab, and 38 out of 45 patients (84.44%) remained free from OCS at 36 months. Only three patients (6.67%) maintained (or increased) the baseline OCS dose, showing no reduction at 36 months (Table 2).

The change in OCS use was also investigated in patients grouped according to their baseline OCS dose (≤5 mg/day or >5 mg/day) (Figure 5 and Table S2). The overall population and the subgroup of patients with a baseline OCS dose >5 mg/day showed a similar OCS reduction pattern, with mean OCS doses of 1.80 (6.02) and 1.88 (5.63) at 36 months, respectively (Figure 5). An almost identical percentage of patients discontinued OCS at 36 months regardless of the baseline OCS dose (85.71% of patients with OCS ≤ 5 mg/day and 83.33% of patients with OCS > 5 mg/day) (Table S2).

### 3.7. Benralizumab Reduced the Need for Asthma Background Medication

The daily ICS dose used by patients decreased progressively over time, from a mean of 1006.93 (402.56) at baseline (*n* = 87) to a mean of 800.04 (394.89) at 36 months (*n* = 70), with an overall reduction of 20.55%. Accordingly, the percentage of patients taking a low dose of ICS (<500 mcg/day) increased from 9.20% at baseline to 32.86% at 36 months, and a significant reduction in the required dose of ICS was observed over time (*p* = 0.0010, Table 3). In particular, the chances of patients requiring a medium ICS dose (≥500 and <1000 mcg/day) at 12, 24, and 36 months were reduced by 78%, 83%, and 89% compared with baseline (0.22 [0.07:0.71], 0.17 [0.06:0.55], and 0.11 [0.02:0.68], respectively), and the chances of requiring a high ICS dose (≥1000 mcg/day) at 6, 12, 24, and 36 months were reduced by 87%, 90%, 91%, and 85% compared with baseline (0.13, 95% CI 0.02:0.77; 0.10, 95% CI 0.02:0.57; 0.09, 95% CI 0.01:0.54), and 0.15 (95% CI 0.05:0.47), respectively) (Table 3). Benralizumab treatment not only lowered the ICS dose but also decreased the need for other asthma medications, such as the use of as-needed relievers SABA or ICS/LABA (*p* < 0.0001), LAMA (*p* = 0.0008), anti LT (*p* < 0.0001), and theophylline (*p* = 0.0070) (Table 3).

### 3.8. Benralizumab Promoted the Achievement of CR

**Figure 6:**
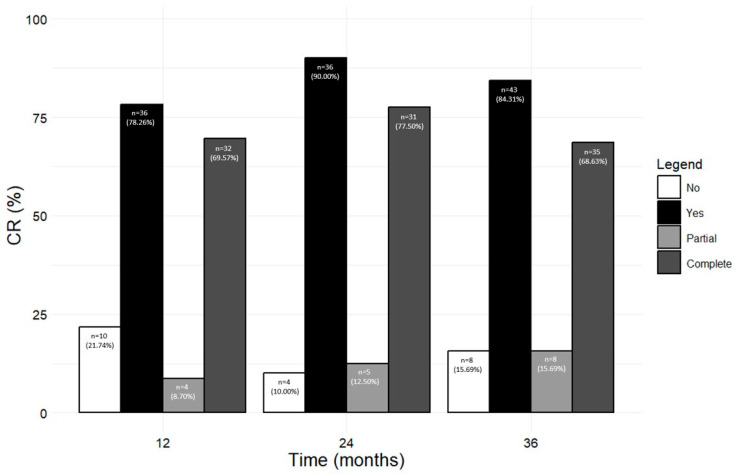
Number and percentage of patients who achieved and did not achieve CR (either pCR or cCR) at 12, 24, and 36 months during the treatment with benralizumab (from a previous time point).

Figure 6 shows the number and percentage of patients who achieved any CR, including pCR and cCR, at 12, 24, and 36 months (from the corresponding previous time point). A total of 36 out of 46 patients (78.26%) reached CR (either pCR, 8.70% or cCR, 69.57%) after 12 months; the rate of CR further increased at the following time points, with 90.00% and 84.31% of patients in CR at 24 and 36 months, respectively. When pCR and cCR were considered separately, the percentage of patients in pCR steadily increased from 8.70% at 12 months to 15.69% at 36 months, while most patients were in cCR from 12 months onwards (69.57% at 12 months, 77.50% at 24 months, and 68.63% at 36 months). The percentage of patients who did not achieve any kind of CR dropped from 21.74% at 12 months to 10.00% and 15.69% at 24 and 36 months, respectively (Figure 6). We also considered the percentage of patients who reached CR from baseline to each time point; as shown in Figure S1, similar results were obtained (at 36 months, 85.71% of patients achieved any CR, with 12.50% patients in pCR and 73.21% patients in cCR).

## 4. Discussion

This study provides a comprehensive analysis of benralizumab effectiveness by retrospectively evaluating a total of 108 Italian patients with SEA treated for up to 36 months. Even though a high number of real-life studies have thus far evaluated the effectiveness of benralizumab on SEA patients, to our knowledge this is the first study conducted on more than 100 SEA patients treated over a period longer than 2 years. The longest real-life study (up to 48 months) was published by Numata and colleagues; however, only 23 SEA patients were initially included and fewer were followed for the entire period [33]. Similarly, Caminati and colleagues evaluated asthma outcomes in 68 mepolizumab-switched patients treated with benralizumab for a median period of 31 months [30]. In a more recent work, Fyles et al. considered a population of 81 SEA patients treated with either mepolizumab or benralizumab for up to 36 months; however, the majority of data were presented for the overall population, and thus, it is not possible to extrapolate the clinical improvements experienced by benralizumab-treated patients for each single outcome [31].

The baseline clinical characteristics reveal a severely compromised SEA patient population. The presence of circulating eosinophils (600 cells/mm^3^), the high AER (3.84), the suboptimal FEV_1_/FVC and FEV_1_ (predicted: 74.50%), and the low scores of various PROs (with a mean ACT score: 14.41) confirm the poor control of the disease. In addition, the high percentages of patients taking OCS and experiencing comorbidities, including CRSwNP in 60.19% patients, bronchiectasis in almost 27% patients, and EGPA in 4.71% patients, further corroborate the high disease burden in our benralizumab-treated population. Nevertheless, the elevated number of comorbidities did not seem to impair the overall effectiveness of the anti-IL-5R. Additionally, benralizumab may also induce favorable outcomes in CRSwNP, bronchiectasis and EGPA; for these conditions, RCTs evaluating benralizumab efficacy are either currently ongoing (NCT04157335, NCT05006573) or terminated (NCT04157348), with positive effects demonstrated in EGPA patients [39].

The results from this long-term study reinforce the remarkable effectiveness of benralizumab in minimizing the number of exacerbations while maintaining minimal BEC throughout the study period. Benralizumab rapidly decreased the frequency of exacerbations, with a reduction in AER that persisted throughout the 36-month period (AER reduction ranging from 89% at 36 months to 95% at 12 months). Although the phenotype of exacerbations was determined in a small subgroup of patients at baseline only, we may speculate that benralizumab reduced all types of exacerbations, both infectious and non-infectious. This hypothesis is supported by (1) the extensive effect seen throughout the treatment period; (2) the high prevalence of bacterial-mediated exacerbations (more than 50%) observed at baseline; and (3) benralizumab novel MoA, which implies a broad modulation of the immune system, including the increased activation of NK cells [26]. Considering the antiviral and antibacterial role exerted by NK cells, it is plausible that their increased function contributed to prevent infections and infectious-related exacerbations in patients during benralizumab treatment. These data demonstrate that the effectiveness of benralizumab in preventing exacerbations is truly long-lasting, and the prominent extent of AER reduction is consistent with previous real-life studies [25,28,29,30].

Although benralizumab was anticipated to have a positive impact on AER over time, variable results have been published regarding its ability to enhance lung function over the long term. In our study, benralizumab significantly increased FEV_1_ over time, with a remarkable +440 mL volume gain and predicted levels reaching normal values up to 36 months. This is the first time that benralizumab has been shown to induce a durable increase in respiratory function in real life up to 3 years; these results are in line with the recently published 96-week data from the ANANKE study, in which both pre-BD FEV_1_ and FVC peaked after 96 weeks of treatment [25], and complement the data obtained from RCTs, where the initial improvement in lung function was stabilized over a two-year period [40]. As already mentioned, the novel MoA of benralizumab postulated by Bergantini et al., which involves the modulation of circulating CD3 + T subsets and increased activation of NK cells, even in the absence of eosinophils, may play a key role in the benralizumab-mediated long-term improvement in lung function [26].

Furthermore, McIntosh et al. found that benralizumab significantly reduced the mucus score in SEA patients treated up to 2.5 years; this result was accompanied by a parallel decrease in airway occlusion and improved ventilation, FEV_1_, and ACQ-6 score [41]. As mucus accumulation has a profound impact on respiratory function in asthma patients, the benralizumab-mediated dissolution of mucus plugs may have contributed to the long-term favorable respiratory outcomes in our study. On the other hand, Numata and colleagues reported a decline in FEV_1_ levels registered after 24 months [33]. In light of the data obtained from our study and other studies, it is possible that this result is biased due to the low number of patients considered in the study. The authors also speculated that the observed FEV_1_ reduction may be caused by an airway obstruction mechanism induced by the long-term administration of a single biological, or a decrease adherence to inhaled therapies, or a physiological decline in pulmonary function [33]. Regardless of the reason justifying the different results, more studies with a greater number of patients are needed to ascertain benralizumab’s long-term effectiveness on lung function and further elucidate the specific mechanism/s leading to increased lung function.

As measured by the ACT questionnaire, asthma control was significantly improved, with a mean ACT score greater than 21 at all timepoints. The significant results obtained in ACQ and AQLQ reinforced the achievement of good asthma control and demonstrated an overall improvement of QoL.

Benralizumab not only induced profound beneficial effects in terms of asthma symptoms but also improved sinonasal symptoms in comorbid patients with CRSwNP, as demonstrated by the significant and progressive changes recorded in VAS and SNOT-22. Although benralizumab currently lacks the indication for the treatment of CRSwNP, growing evidence indicates that the anti-IL-5R has a positive impact on nasal symptoms in comorbid patients [42,43,44,45]. Notably, a recent work by Santomasi and colleagues showed that benralizumab was not only effective in decreasing the SNOT-22 score, but it also significantly reduced the nasal polyps score (NPS), determined by nasal endoscopy, and the number of nasal eosinophils and neutrophils, assessed via nasal cytology, in SEA patients with CRSwNP [45]. Collectively, these data corroborate the theory of “united airway disease” [46], implying that SEA and CRSwNP share the same eosinophilic-driven pathophysiology in comorbid patients, and benralizumab could indeed represent the optimal therapeutic strategy to tackle both pathologies simultaneously.

The marked OCS-sparing effect of benralizumab is well recognized. In the pivotal PONENTE study, almost 63% of patients completely eliminated the use of OCS and more than 80% of patients either eliminated OCS or maintained a minimum dose due to adrenal insufficiency [47]. Similarly, the OCS dose has been either reduced or zeroed in real-life studies where patients were treated for periods longer than one year [25,29,30,33]. Our data indicate that benralizumab induced a durable OCS reduction, and this effect further increased over time. Indeed, the minimal mean dose of OCS was registered at 36 months; at this time point, almost the totality of patients (93.33%) successfully decreased their OCS dose by any extent, and 84.44% patients permanently discontinued OCS therapy. To our knowledge, these percentages are the highest ever recorded in the literature. The sub-analysis conducted on patients requiring a daily OCS dose of either ≤ or >5 mg substantiated the findings from the PONENTE trial, which showed that benralizumab OCS-sparing effect, was independent of the baseline dose [47].

Beyond the high rate of OCS reduction and elimination, benralizumab treatment was associated with a net and significant decrease in the dose of the maintenance ICS dose, with an overall reduction of 20.55% and a decreased probability of requiring medium and high ICS doses over time. Consistently, there were more than 20% patients transitioning from medium or high ICS doses to low ICS doses (<500 mcg/day) at 36 months. We also observed a progressive decline in the use of all other asthma therapies, including LAMA, anti-LT, theophylline, and as needed SABA and/or ICS/LABA. Notably, patients could reduce all these medications while maintaining good asthma control, as demonstrated by ACT and ACQ scores. To date, the effect of benralizumab on asthma medication other than OCS has not been extensively investigated. In the RCT SHAMAL, up to 92% of patients successfully reduced their high-dose ICS and 96% maintained such reductions up to 48 weeks without compromising asthma control (87% of patients were free from exacerbations by week 48) [34]. In real life, 66.3% of patients decreased the use of maintenance medications (ICS dose reduction and/or LABA, LAMA montelukast interruption) over a mean treatment period of 19.7 months. Similarly to our data, approximately 25% of patients reduced the ICS dose [29]. Given the extremely poor adherence to inhaled therapy in SA patients [48], benralizumab-mediated reduction in ICS and other background therapies may provide further beneficial effects by leading to (1) higher compliance to inhalers; (2) better asthma control in patients who do not strictly adhere to inhaled therapy regimen.

Since the advent of biological therapies for asthma and the compelling amelioration of patients’ symptoms, the achievement of CR in SA patients has become possible. However, the criteria defining CR used in the various studies published so far have been somehow arbitrary. Recently, a Delphi consensus reached by members of the SANI study group agreed on the criteria to identify patients in pCR and cCR [19]. Based on these criteria, our results show that CR, and specifically cCR, was achieved by more than the half of the patients at all time points considered (up to 90.00% patients in CR at 24 months, of which 77.50% patients were in cCR). These data mean that benralizumab could permanently eradicate the disease in the vast majority of patients during the three-year study duration. CR was evaluated in previous studies, both RCT and in real life; the results show that benralizumab induced CR in percentages of SEA patients ranging from 14.5% (in the SIROCCO and CALIMA RCTs) [49] to 43% (in the real-world XALOC-1 study [50]). Compared to the latter studies, a slightly higher percentage of patients (54%) reached clinical remission at 48 weeks in the SHAMAL RCT despite the reduction in background ICS dose [34].

The percentage of patients achieving CR in our study seems to exceed the results previously reported; however, attention should be paid to the criteria employed to define CR, as they vary across the studies, and they differ from the criteria used herein. For instance, in the XALOC-1 study, the percentage of patients reaching CR (43%) was calculated without including any respiratory parameters and considering an ACT score ≥16 points [50]. Importantly, Campisi et al. found that SEA patients achieved CR more frequently in the absence of bronchiectasis [51], suggesting a negative impact of this comorbidity on benralizumab effectiveness [51]. The inclusion of a variable proportion of patients with this comorbidity, accompanied by the methodological differences used across the studies, may justify the variable results obtained so far. In our study, CR was evaluated in all patients, including those with bronchiectasis (which affected approximately a quarter of our patient population). The strict criteria employed in our study to define cCR and the inclusion of patients with bronchiectasis add further value to the rates of cCR reported here, which are unprecedented but justified by the striking effect of benralizumab observed in all the single outcomes (exacerbations, lung function, asthma control and OCS use). The long-term implications of achieving clinical remission through benralizumab treatment could encompass a better prognosis and extended life expectancy in SEA patients. These aspects are even more relevant considering the increasing prevalence and severity of late-onset asthma, and SEA in particular, as the global population ages [52].

Drawing upon the notion of “deep remission” related to rheumatoid arthritis, Oishi and colleagues also assessed deep remission in SA patients by considering the successful inhibition of T2 inflammation with BEC < 300 cells/mm^3^ and FeNO < 35 ppb or < 50 ppb. [53,54]. While we did not formally assess the rate of patients achieving deep remission in our study, we anticipate a similar rate to those who achieved CR, owing to the extensive drop in BEC specifically induced by benralizumab MoA.

The retrospective design of this study represents its main limitation, as it is associated with a considerable loss of data during the 36-month treatment period. In general, a certain degree of missing data is anticipated in real-life, and retrospective multicentric studies can be attributed to several factors, including (1) variability in the collection of certain parameters across different centers, (2) loss of patients at follow-up due to various reasons (e.g., patient relocation, change in healthcare provider, etc.), and (3) data recording and collection from medical charts (not originally intended for research purposes) [55]. In our case, the extensive study duration and the monitoring of patients amid the COVID-19 pandemic also negatively affected patients’ attendance at follow-up visits, further contributing to the loss of data during the observation period. Adverse events were not collected because the study’s main purpose was to evaluate the effectiveness of benralizumab and this may represent another limitation. As already mentioned above, additional studies will be needed to validate our results over even longer treatment periods and by considering greater numbers of patients. Ideally, future studies will be conducted with a prospective design to limit the loss of data. A thorough evaluation of safety would also be valuable to confirm the long-term safety of benralizumab already observed in the MELTEMI study [27].

## 5. Conclusions

This study offers a comprehensive assessment of benralizumab long-term effectiveness on SEA by examining a meaningful sample population at various time points, up to 36 months of treatment. These impressive data not only comprehensively illustrate the long-lasting response to benralizumab in all the considered asthma clinical outcomes, but also reveal the simultaneous positive effects on CRSwNP symptoms. The large percentage of patients who reached either pCR or cCR is indicative of the long-term well-being induced by benralizumab and support its role as a disease-modifying anti-asthmatic therapy for the management of SEA. More research should corroborate these results, with a focus on minimizing data loss and exploring further facets of benralizumab MoA and effectiveness, including the impact on adherence to inhaled therapy, airway remodeling, and the achievement of deep remission.

## Figures and Tables

**Table 1:** Demographic, social, and clinical characteristics of patient population at baseline. Data refer to *n* = 108 patients, unless otherwise specified, and are expressed as the number of subjects (percentage), mean (SD), or median (range) as appropriate.

Characteristics at Baseline	Patient Population
Age (years)	55.96 (11.38)
Sex (female)	64 (59.26%)
BMI (kg/m^2^)	
Underweight/normal weight	46 (42.59%)
Overweight	46 (42.59%)
Obese	16 (14.81%)
Smoking status	
Non-smokers	78 (72.22%)
Previous smokers	29 (26.85%)
Current smokers	1 (0.93%)
Comorbidities	94 (87.04%)
CRS	67 (62.62%)
CRSwNP	65 (60.19%)
Allergic rhinitis	57 (52.78%)
Gastroesophageal reflux disease (GERD)	39 (36.11%)
Bronchiectasis (*n* = 104)	28 (26.96%)
EGPA (*n* = 96)	4 (4.71%)
Eosinophilic esophagitis (*n* = 93)	1 (1.08%)
Age at asthma diagnosis (years) (*n* = 106)	35.68 (15.95)
ICS dose (mcg/day) (*n* = 87)	1006.93 (402.56)
<500	8 (9.20%)
≥500 and <1000	44 (50.57%)
≥1000	35 (40.23%)
LAMA (*n* = 107)	79 (73.83%)
Anti LT (*n* = 107)	52 (48.60%)
Theophylline (*n* = 106)	6 (5.66%)
SABA and/or ICS/LABA as needed (times per day) (*n* = 88)	3.10 (4.19)
OCS	67 (62.04%)
OCS dose (prednisone equivalent) (mg/day) (*n* = 67)	7.95 (7.42)
≤5 mg/day	26 (38.81%)
>5 mg/day	41 (61.19%)
Biologic switch (*n* = 98)	
Naïve	89 (82.41%)
Switched	19 (17.59%)
BEC (cells/mm^3^) (*n* = 104)	600 (28–3350)
FeNO (ppb) (N = 55)	61.95 (55.66)
Total serum IgE (IU/mL) (*n* = 91)	209 (4.9–1940)
AER (*n* = 107)	3.84 (3.18)
Exacerbation phenotype (*n* = 45)	
Bacterial	24 (53.33%)
Viral	7 (15.56%)
Non-infectious	14 (31.11%)
Lung function	
FEV_1_ (L/min) (*n* = 101)	1.94 (0.83)
FVC (L) (*n* = 90)	3.30 (1.00)
FEV_1_ (% pred.) (*n* = 86)	74.50 (20.68)
FVC (% pred.) (*n* = 91)	93.97 (20.73)
FEV_1_/FVC (*n* = 91)	54.11 (24.87)
Asthma PROs	
ACT score (*n* = 104)	14.41 (5.08)
ACQ score (*n* = 39)	2.33 (1.25)
AQLQ score (*n* = 31)	3.76 (1.45)
CRSwNP PROs	
VAS (*n* = 60)	5.14 (3.13)
SNOT-22 score (*n* = 57)	47.21 (20.67)

**Table 2:** Extent of OCS dose reduction achieved by patients during the treatment with benralizumab. Data are expressed as *n* (%).

Extent of OCS Reduction	Month 6 (*n* = 64)	Month 12 (*n* = 47)	Month 24 (*n* = 43)	Month 36 (*n* = 45)
Any reduction	52 (81.25%)	41 (87.23%)	39 (90.7%)	42 (93.33%)
≥90%	39 (60.94%)	34 (72.34%)	34 (79.07%)	38 (84.44%)
≥75%	42 (65.62%)	38 (80.85%)	37 (86.05%)	39 (86.67%)
≥50%	50 (78.12%)	41 (87.23%)	38 (88.37%)	42 (93.33%)
≥25%	51 (79.69%)	41 (87.23%)	39 (90.7%)	42 (93.33%)
No reduction	12 (18.75%)	6 (12.77%)	4 (9.3%)	3 (6.67%)
Elimination	39 (60.94%)	34 (72.34%)	33 (76.74%)	38 (84.44%)

**Table 3:** Change in asthma medication use during the treatment with benralizumab. Descriptive statistics with a summary output of mixed-model on asthma medications other than OCS (ICS, SABA or ICS-LABA as needed, LAMA, anti LT, and theophylline) recorded at baseline and during the treatment with benralizumab. Mean (SD) and *n* values or *n* (percentage) and the beta regression coefficient (or OR where appropriate) with 95% CI are reported for each time point.

Parameter	Baseline	Month 6	Month 12	Month 24	Month 36	*p*-Value
ICS dose (mcg/day)	1006.93 (402.56) *n* = 87	895.09 (420.65) *n* = 85	853.93 (408.02) *n* = 68	841.47 (411.64) *n* = 70	800.04 (394.89) *n* = 70	
ICS dose (mcg/day)	1006.93 (402.56)	895.09 (420.65)	853.93 (408.02)	841.47 (411.64)	800.04 (394.89)	0.0010 †
<500	8 (9.20%)	17 (20.00%)	19 (27.94%)	21 (30.00%)	23 (32.86%)	
≥500 and <1000	44 (50.57%)	43 (50.59%)	32 (47.06%)	32 (45.71%)	31 (44.29%)	
≥1000	35 (40.23%)	25 (29.41%)	17 (25.00%)	17 (24.29%)	16 (22.86%)	
*	1	0.37 (0.12:1.13)	0.22 (0.07:0.71)	0.17 (0.06:0.55)	0.11 (0.02:0.68)	
#	1	0.13 (0.02:0.77)	0.10 (0.02:0.57)	0.09 (0.01:0.54)	0.15 (0.05:0.47)	
SABA or ICS-LABA as needed (times per day)	3.10 (4.19)	0.35 (0.96)	0.2 (0.6)	0.43 (1.42)	0.38 (1.85)	<0.0001
	0	−2.72 (−3.37:−2.07)	−2.78 (−3.51:−2.06)	−2.59 (−3.3:−1.89)	−2.73 (−3.43:−2.04)	
	*n* = 88	*n* = 85	*n* = 61	*n* = 67	*n* = 71	
LAMA						0.0008
No	28 (26.17%)	29 (28.16%)	28 (29.17%)	34 (40%)	38 (44.19%)	
Yes	79 (73.83%)	74 (71.84%)	68 (70.83%)	51 (60%)	48 (55.81%)	
	1	0.50 (0.13:1.85)	0.29 (0.07:1.12)	0.13 (0.03:0.53)	0.04 (0.01:0.20)	
	*n* = 107	*n* = 103	*n* = 96	*n* = 85	*n* = 86	
Anti LT						<0.0001
No	55 (51.40%)	56 (54.9%)	55 (57.89%)	57 (67.06%)	59 (68.6%)	
Yes	52 (48.60%)	46 (45.10%)	40 (42.11%)	28 (32.94%)	27 (31.4%)	
	1	0.48 (0.16:1.40)	0.22 (0.07:0.70)	0.06 (0.02:0.23)	0.06 (0.02:0.23)	
	*n* = 107	*n* = 102	*n* = 95	*n* = 85	*n* = 86	
Theophylline						0.0070
No	100 (94.34%)	100 (99.01%)	95 (98.96%)	82 (96.47%)	84 (100%)	
Yes	6 (5.66%)	1 (0.99%)	1 (1.04%)	3 (3.53%)	0 (0%)	
	1	0.01 (0:0.68)	0.01 (0:0.38)	0.08 (0.01:1.14)	0 (0:Inf)	
	*n* = 106	*n* = 101	*n* = 96	*n* = 85	*n* = 84	

† *p* value estimated by fitting a multinomial log-linear model corrected for center and patients variability * ICS dose contrast, ≥500 and <1000 mcg/day versus <500 mcg/day; # ICS dose contrast, ≥500 1000 and <1000 mcg/day versus <500 mcg/day.

## Data Availability

The data presented in this study are available upon reasonable request from the corresponding author (Laura Pini, laura.pini@unibs.it).

## Supplementary Materials

The following supporting information can be downloaded at: https://www.mdpi.com/article/10.3390/jcm13103013/s1, List of additional members of SANI. Table S1: Descriptive statistics with a summary output of a mixed model on FeNO and lung function parameters recorded at baseline and during the treatment with benralizumab (*n* = 108 unless otherwise specified). Mean (SD) and *n* values or *n* (percentage) and beta regression coefficients with 95% CI are reported for each time point. Table S2: Extent of OCS dose reduction during treatment with benralizumab in patients grouped according to OCS dose at baseline (≤5 mg/day or >5 mg/day). Figure S1: Number and percentage of patients who achieved and did not achieve CR (either pCR or cCR) at 12, 24, and 36 months during the treatment with benralizumab (values calculated from baseline).

## References

[1] Chung K.F., Wenzel S.E., Brozek J.L., Bush A., Castro M., Sterk P.J., Adcock I.M., Bateman E.D., Bel E.H., Bleecker E.R. (2014). International ERS/ATS guidelines on definition, evaluation and treatment of severe asthma. Eur. Respir. J..

[2] Hekking P.-P.W., Wener R.R., Amelink M., Zwinderman A.H., Bouvy M.L., Bel E.H. (2015). The prevalence of severe refractory asthma. J. Allergy Clin. Immunol..

[3] Bagnasco D., Paggiaro P., Latorre M., Folli C., Testino E., Bassi A., Milanese M., Heffler E., Manfredi A., Riccio A.M. (2021). Severe asthma: One disease and multiple definitions. World Allergy Organ. J..

[4] Buhl R., Humbert M., Bjermer L., Chanez P., Heaney L.G., Pavord I., Quirce S., Virchow J.C., Holgate S. (2017). Severe eosinophilic asthma: A roadmap to consensus. Eur. Respir. J..

[5] Perez-de-Llano L., Tran T.N., Al-ahmad M., Alacqua M., Bulathsinhala L., Busby J., Canonica G.W., Carter V., Chaudhry I., Christoff G.C. (2020). Characterization of Eosinophilic and Non-Eosinophilic Severe Asthma Phenotypes and Proportion of Patients with These Phenotypes in the International Severe Asthma Registry (ISAR). C21. Advances in Adult and Pediatric Asthma Phenotyping and Endotyping.

[6] Maio S., Baldacci S., Bresciani M., Simoni M., Latorre M., Murgia N., Spinozzi F., Braschi M., Antonicelli L., Brunetto B. (2018). RItA: The Italian severe/uncontrolled asthma registry. Allergy.

[7] Bakakos A., Loukides S., Bakakos P. (2019). Severe Eosinophilic Asthma. J. Clin. Med..

[8] de Groot J.C., Ten Brinke A., Bel E.H.D. (2015). Management of the patient with eosinophilic asthma: A new era begins. ERJ Open Res..

[9] Heaney L.G., Perez de Llano L., Al-Ahmad M., Backer V., Busby J., Canonica G.W., Christoff G.C., Cosio B.G., FitzGerald J.M., Heffler E. (2021). Eosinophilic and Noneosinophilic Asthma: An Expert Consensus Framework to Characterize Phenotypes in a Global Real-Life Severe Asthma Cohort. Chest.

[10] Laidlaw T.M., Mullol J., Woessner K.M., Amin N., Mannent L.P. (2021). Chronic Rhinosinusitis with Nasal Polyps and Asthma. J. Allergy Clin. Immunol. Pract..

[11] Massoth L., Anderson C., McKinney K.A. (2019). Asthma and Chronic Rhinosinusitis: Diagnosis and Medical Management. Med. Sci..

[12] 2023 Gina Main Report. https://ginasthma.org/wp-content/uploads/2023/05/GINA-2023-Full-Report-2023-WMS.pdf.

[13] Price D.B., Trudo F., Voorham J., Xu X., Kerkhof M., Ling Zhi Jie J., Tran T.N. (2018). Adverse outcomes from initiation of systemic corticosteroids for asthma: Long-term observational study. J. Asthma Allergy.

[14] Lee H., Ryu J., Nam E., Chung S.J., Yeo Y., Park D.W., Park T.S., Moon J.-Y., Kim T.-H., Sohn J.W. (2019). Increased mortality in patients with corticosteroid-dependent asthma: A nationwide population-based study. Eur. Respir. J..

[15] Bourdin A., Molinari N., Vachier I., Pahus L., Suehs C., Chanez P. (2017). Mortality: A neglected outcome in OCS-treated severe asthma. Eur. Respir. J..

[16] Lommatzsch M., Brusselle G.G., Levy M.L., Canonica G.W., Pavord I.D., Schatz M., Virchow J.C. (2023). A2BCD: A concise guide for asthma management. Lancet Respir. Med..

[17] Chen W., Tran T.N., Sadatsafavi M., Murray R., Wong N.C.B., Ali N., Ariti C., Bulathsinhala L., Gil E.G., FitzGerald J.M. (2023). Impact of Initiating Biologics in Patients With Severe Asthma on Long-Term Oral Corticosteroids or Frequent Rescue Steroids (GLITTER): Data From the International Severe Asthma Registry. J. Allergy Clin. Immunol. Pract..

[18] Chen M., Shepard K., Yang M., Raut P., Pazwash H., Holweg C.T.J., Choo E. (2021). Overlap of allergic, eosinophilic and type 2 inflammatory subtypes in moderate-to-severe asthma. Clin. Exp. Allergy J. Br. Soc. Allergy Clin. Immunol..

[19] Canonica G.W., Blasi F., Carpagnano G.E., Guida G., Heffler E., Paggiaro P., Allegrini C., Antonelli A., Aruanno A., Bacci E. (2023). Severe Asthma Network Italy Definition of Clinical Remission in Severe Asthma: A Delphi Consensus. J. Allergy Clin. Immunol. Pract..

[20] Lommatzsch M., Brusselle G.G., Canonica G.W., Jackson D.J., Nair P., Buhl R., Virchow J.C. (2022). Disease-modifying anti-asthmatic drugs. Lancet.

[21] https://www.ema.europa.eu/en/documents/product-information/fasenra-epar-product-information_en.pdf.

[22] Dagher R., Kumar V., Copenhaver A.M., Gallagher S., Ghaedi M., Boyd J., Newbold P., Humbles A.A., Kolbeck R. (2022). Novel mechanisms of action contributing to benralizumab’s potent anti-eosinophilic activity. Eur. Respir. J..

[23] Caminati M., Bagnasco D., Vaia R., Senna G. (2019). New horizons for the treatment of severe, eosinophilic asthma: Benralizumab, a novel precision biologic. Biol. Targets Ther..

[24] Menzella F., Biava M., Bagnasco D., Galeone C., Simonazzi A., Ruggiero P., Facciolongo N. (2019). Efficacy and steroid-sparing effect of benralizumab: Has it an advantage over its competitors?. Drugs Context.

[25] Vultaggio A., Aliani M., Altieri E., Bracciale P., Brussino L., Caiaffa M.F., Cameli P., Canonica G.W., Caruso C., Centanni S. (2023). Long-term effectiveness of benralizumab in severe eosinophilic asthma patients treated for 96-weeks: Data from the ANANKE study. Respir. Res..

[26] Bergantini L., d’Alessandro M., Pianigiani T., Cekorja B., Bargagli E., Cameli P. (2023). Benralizumab affects NK cell maturation and proliferation in severe asthmatic patients. Clin. Immunol..

[27] Korn S., Bourdin A., Chupp G., Cosio B.G., Arbetter D., Shah M., Gil E.G. (2021). Integrated Safety and Efficacy Among Patients Receiving Benralizumab for Up to 5 Years. J. Allergy Clin. Immunol. Pract..

[28] Vitale C., Maglio A., Pelaia C., D’Amato M., Ciampo L., Pelaia G., Molino A., Vatrella A. (2023). Effectiveness of Benralizumab in OCS-Dependent Severe Asthma: The Impact of 2 Years of Therapy in a Real-Life Setting. J. Clin. Med..

[29] Sposato B., Scalese M., Camiciottoli G., Carpagnano G.E., Pelaia C., Santus P., Pelaia G., Palmiero G., Di Tomassi M., Ronchi M.C. (2022). Severe asthma and long-term Benralizumab effectiveness in real-life. Eur. Rev. Med. Pharmacol. Sci..

[30] Caminati M., Marcon A., Guarnieri G., Miotti J., Bagnasco D., Carpagnano G.E., Pelaia G., Vaia R., Maule M., Vianello A. (2023). Benralizumab Efficacy in Late Non-Responders to Mepolizumab and Variables Associated with Occurrence of Switching: A Real-Word Perspective. J. Clin. Med..

[31] Fyles F., Nuttall A., Joplin H., Burhan H. (2023). Long-Term Real-World Outcomes of Mepolizumab and Benralizumab Among Biologic-Naive Patients With Severe Eosinophilic Asthma: Experience of 3 Years’ Therapy. J. Allergy Clin. Immunol. Pract..

[32] Risco M., Sotomayor J., Alvarez-Sala P., Piorno I., Diaz-Campos R., Moya B., Crespo J.F., Fernandez C., GarcÃa Moguel I. (2022). Long-term effectiveness and safety of benralizumab for uncontrolled eosinophilic asthma in real-word practice. J. Allergy Clin. Immunol..

[33] Numata T., Araya J., Okuda K., Miyagawa H., Minagawa S., Ishikawa T., Hara H., Kuwano K. (2022). Long-Term Efficacy and Clinical Remission After Benralizumab Treatment in Patients with Severe Eosinophilic Asthma: A Retrospective Study. J. Asthma Allergy.

[34] Jackson D.J., Heaney L.G., Humbert M., Kent B.D., Shavit A., Hiljemark L., Olinger L., Cohen D., Menzies-Gow A., Korn S. (2024). Reduction of daily maintenance inhaled corticosteroids in patients with severe eosinophilic asthma treated with benralizumab (SHAMAL): A randomised, multicentre, open-label, phase 4 study. Lancet.

[35] Piano Terapeutico AIFA per la Prescrizione SSN di Fasenra (Benralizumab) Nell’asma Grave Eosinofilico Refrattario [Internet]. Gazzetta Ufficiale della Repubblica Italiana. https://www.gazzettaufficiale.it/atto/serie_generale/caricaDettaglioAtto/originario?atto.dataPubblicazioneGazzetta=2019-02-12&atto.codiceRedazionale=19A00829&elenco30giorni=false.

[36] Gelman A., Hill J. (2007). Data Analysis Using Regression and Multilevel/Hierarchical Models.

[37] https://www.r-project.org/.

[38] Toma S., Hopkins C. (2016). Stratification of SNOT-22 scores into mild, moderate or severe and relationship with other subjective instruments. Rhinol. J..

[39] Wechsler M.E., Nair P., Terrier B., Walz B., Bourdin A., Jayne D.R.W., Jackson D.J., Roufosse F., Börjesson Sjö L., Fan Y. (2024). Benralizumab versus Mepolizumab for Eosinophilic Granulomatosis with Polyangiitis. N. Engl. J. Med..

[40] FitzGerald J.M., Bleecker E.R., Bourdin A., Busse W.W., Ferguson G.T., Brooks L., Barker P., Martin U.J. (2019). Two-Year Integrated Efficacy And Safety Analysis Of Benralizumab In Severe Asthma. J. Asthma Allergy.

[41] McIntosh M.J., Kooner H.K., Eddy R.L., Wilson A., Serajeddini H., Bhalla A., Licskai C., Mackenzie C.A., Yamashita C., Parraga G. (2023). CT Mucus Score and 129Xe MRI Ventilation Defects after 2.5 Years’ Anti-IL-5Rα in Eosinophilic Asthma. CHEST.

[42] Nolasco S., Crimi C., Pelaia C., Benfante A., Caiaffa M.F., Calabrese C., Carpagnano G.E., Ciotta D., D’Amato M., Macchia L. (2021). Benralizumab Effectiveness in Severe Eosinophilic Asthma with and without Chronic Rhinosinusitis with Nasal Polyps: A Real-World Multicenter Study. J. Allergy Clin. Immunol. Pract..

[43] Lombardo N., Pelaia C., Ciriolo M., Della Corte M., Piazzetta G., Lobello N., Viola P., Pelaia G. (2020). Real-life effects of benralizumab on allergic chronic rhinosinusitis and nasal polyposis associated with severe asthma. Int. J. Immunopathol. Pharmacol..

[44] Bagnasco D., Brussino L., Bonavia M., Calzolari E., Caminati M., Caruso C., D’Amato M., De Ferrari L., Di Marco F., Imeri G. (2020). Efficacy of Benralizumab in severe asthma in real life and focus on nasal polyposis. Respir. Med..

[45] Santomasi C., Buonamico E., Dragonieri S., Iannuzzi L., Portacci A., Quaranta N., Carpagnano G.E. (2023). Effects of benralizumab in a population of patients affected by severe eosinophilic asthma and chronic rhinosinusitis with nasal polyps: A real life study. Acta Biomed. Atenei Parm..

[46] Kanda A., Kobayashi Y., Asako M., Tomoda K., Kawauchi H., Iwai H. (2019). Regulation of Interaction between the Upper and Lower Airways in United Airway Disease. Med. Sci..

[47] Menzies-Gow A., Gurnell M., Heaney L.G., Corren J., Bel E.H., Maspero J., Harrison T., Jackson D.J., Price D., Lugogo N. (2022). Oral corticosteroid elimination via a personalised reduction algorithm in adults with severe, eosinophilic asthma treated with benralizumab (PONENTE): A multicentre, open-label, single-arm study. Lancet Respir. Med..

[48] Caminati M., Vianello A., Andretta M., Menti A.M., Tognella S., Esposti L.D., Gianenrico S. (2020). Astonishing low adherence to inhaled therapy characterizes patients with severe asthma treated with biologics. World Allergy Organ. J..

[49] Menzies-Gow A., Hoyte F.L., Price D.B., Cohen D., Barker P., Kreindler J., Jison M., Brooks C.L., Papeleu P., Katial R. (2022). Clinical Remission in Severe Asthma: A Pooled Post Hoc Analysis of the Patient Journey with Benralizumab. Adv. Ther..

[50] Jackson D., Pelaia G., Padilla-Galo A., Watt M., Kayaniyil S., Boarino S., Tena J.S., Shih V., Tran T., Arbetter D. (2023). Asthma Clinical Remission with Benralizumab in an Integrated Analysis of the Real-World XALOC-1 Study. J. Allergy Clin. Immunol..

[51] Campisi R., Nolasco S., Pelaia C., Impellizzeri P., D’Amato M., Portacci A., Ricciardi L., Scioscia G., Crimi N., Scichilone N. (2023). Benralizumab Effectiveness in Severe Eosinophilic Asthma with Co-Presence of Bronchiectasis: A Real-World Multicentre Observational Study. J. Clin. Med..

[52] Hirano T., Matsunaga K. (2018). Late-onset asthma: Current perspectives. J. Asthma Allergy.

[53] Oishi K., Hamada K., Murata Y., Yamaji Y., Asami M., Edakuni N., Hirano T., Matsunaga K. (2022). Achievement rate and predictive factors of the deep remission to biologics in severe asthma. Eur. Respir. J..

[54] Oishi K., Hamada K., Murata Y., Matsuda K., Ohata S., Yamaji Y., Asami-Noyama M., Edakuni N., Kakugawa T., Hirano T. (2023). A Real-World Study of Achievement Rate and Predictive Factors of Clinical and Deep Remission to Biologics in Patients with Severe Asthma. J. Clin. Med..

[55] Talari K., Goyal M. (2020). Retrospective Studies–Utility and Caveats. J. R. Coll. Physicians Edinb..

