# Supplementary material for: Unlocking the Long-Term Effectiveness of Benralizumab in Severe Eosinophilic Asthma: A Three-Year Real-Life Study
Source: J Clin Med. 2024 May 20;13(10):3013. doi: 10.3390/jcm13103013 (PMC11122375; doi:10.3390/jcm13103013)

List of additional members of SANI

Luisa Brussino[1], Cecilia Calabrese[2], Gianna Camiciottoli[3], Giovanna Elisiana Carpagnano[4], Stefano Centanni[5], Angelo Guido Corsico[6], Maria Teresa Costantino[7], Alice D'Adda[8], Simona D'Alo[9], Maria D'Amato[10], Stefano Del Giacco[11], Fabiano Di Marco[12], Nicola Cosimo Facciolongo[13], Eustachio Nettis[14], Eleonora Nucera[15,16], Giovanni Passalacqua[17], Girolamo Pelaia[18], Luisa Ricciardi[19], Luca Richeldi[20], Erminia Ridolo[21], Pierachille Santus[22], Nicola Scichilone[23], Giulia Scioscia[24], Giuseppe Spadaro[25], Paolo Tarsia[26]

[1]Dipartimento di Scienze Mediche, Università degli Studi di Torino; SCDU Immunologia e Allergologia, AO Ordine Mauriziano Umberto I, Torino, Italy
[2] Department of Translational Medical Sciences, University of Campania "Luigi Vanvitelli", Naples, Italy
[3] UNIT ASMA GRAVE - Ambulatorio Asma Grave Pneumologia e Fisiopatologia Toraco-Polmonare, Azienda Ospedaliera Universitaria Careggi (FI), Florence, Italy
[4] Department of Translational Biomedicine and Neuroscience"DiBraiN", University of Bari Aldo Moro, Bari, Italy
[5] Respiratory Unit, ASST Santi Paolo e Carlo, San Paolo Hospital, Department of Health Sciences, University of Milan, Milan, Italy
[6] Division of Respiratory Diseases, IRCCS Policlinico San Matteo, Foundation and Department of Internal Medicine and Therapeutics, University of Pavia, Italy
[7] Allergy and Clinical Immunology Unit, Department of Medicine, "Carlo Poma" Hospital Mantova, Italy
[8] Broncopneumologia, Fondazione IRCCS Ca' Granda Ospedale Maggiore Policlinico, Milan, Italy
[9] UOC Allergologia - PO Civitanova Marche - AREA VASTA 3, Civitanova Marche, Italy
[10] Department of Respiratory Medicine, University "Federico II" of Naples, Italy
[11] Unit of Allergy and Clinical Immunology, Department of Medical Sciences and Public Health, University of Cagliari, Cagliari, Italy
[12] Pulmonary Medicine Unit, ASST Papa Giovanni XXIII Hospital, Bergamo, Italy
[13] UOC di Pneumologia, Arcispedale S. Maria Nuova Azienda USL di Reggio Emilia, Italy
[14] Department of Emergency and Organ Transplantation, School and Chair of Allergology and Clinical Immunology, University of Bari - Aldo Moro, Bari, Italy
[15] Fondazione Policlinico Universitario Agostino Gemelli IRCCS - Università Cattolica del Sacro Cuore Roma, Italy
[16] UOSD Allergologia e Immunologia Clinica, Dipartimento Scienze Mediche e Chirurgiche, Fondazione Policlinico Universitario A. Gemelli IRCCS - Università Cattolica del Sacro Cuore Roma, Italy
[17] Clinica di Malattie Respiratorie e Allergologia, Dip. Medicina Interna, Univ. degli Studi di Genova, IRCCS- AOU San Martino, Genova, Italy
[18] U.O. Malattie dell'Apparato Respiratorio, A.O.U. Mater Domini, Catanzaro, Italy
[19] Allergologia e Immunologia Clinica, AOU Policlinico G. Martino - Università di Messina, Messina, Italy
[20] Fondazione Policlinico Universitario A. Gemelli, IRCCS Catholic University of Rome, Italy
[21] Department of Medicine and Surgery, University of Parma, Parma, Italy
[22] Department of Clinical and Biomedical Sciences, University of Milan, Respiratory Diseases, Sacco University Hospital, ASST Fatebenefratelli-Sacco, Milan, Italy
[23] U.O.C. Pneumologia, Azienda Ospedaliera Universitaria Policlinico P. Giaccone di Palermo, Italy
[24] Malattie Apparato Respiratorio, Dipartimenti delle funzioni Mediche e Sanitarie, Azienda Ospedaliero Universitaria - Ospedali Riuniti, Foggia, Italy
[25] Center for Basic and Clinical Immunology Research (CISI), University of Naples Federico II, Naples, Italy
[26] Pneumology Unit, Grande Ospedale Metropolitano Niguarda, Milan, Italy

Table S1: Descriptive statistics with a summary output of a mixed model on FeNO and lung function parameters recorded at baseline and during the treatment with benralizumab (n=108 unless otherwise specified). Mean (SD) and n values or n (percentage) and beta regression coefficients with 95% CI are reported for each time point.

Parameter	Baseline	Month 6	Month 12	Month 24	Month 36	p-value
**FeNO**	61.95 (55.66)<br>0<br>n=55	28.74 (21.06)<br>-29.69 (-40.63:-18.75)<br>n=49	32.22 (20.23)<br>-25.27 (-37.73:-12.8<br>n=35	41.25 (36.92)<br>-20.2 (-32.82:-7.57)<br>n=33	42.27 (32.31)<br>-18.21 (-30.01:-6.41)<br>n=40	0.0001
**FCV predicted (%)**	93.97 (20.73)<br>0<br>n=91	99.92 (20.58)<br>6.57 (2.99:10.14)<br>n=80	102.26 (24.52)<br>9.29 (5.49:13.08)<br>n=67	100.19 (19.42)<br>8.84 (4.98:12.71)<br>n=64	101.59 (19.38)<br>8.34 (4.69:11.98)<br>n=76	<0.0001
**Post BD FEV_1 (L/min.)**	2.27 (0.72)<br>0<br>61	2.55 (0.77)<br>0.21 (0.08:0.34)<br>42	2.80 (0.74)<br>0.25 (0.09:0.40)	2.65 (0.87)<br>0.14 (-0.01:0.28)<br>27	2.54 (0.96)<br>0.16 (0.02:0.30)<br>37	0.0397
**Post BD FEV1 predicted (%)**	87.13 (21.72)<br>0<br>n=63	93.97 (23.48)<br>7.34 (1.51:13.17)<br>n=42	99.11 (17.11)<br>9.72 (2.87:16.58)<br>n=27	96.8 (20.46)<br>7.8 (1.43:14.17)<br>n=35	92.97 (25.95)<br>6.42 (0.3:12.53)<br>n=38	0.1703
**Post-BD FCV predicted (%)**	87.06 (36.79)<br>0<br>n=60	102.9 (23.35)<br>14.03 (3.28:24.79)<br>n=40	107.93 (21.28)<br>16.26 (3.83:28.68)<br>n=27	104.08 (31.68)<br>11.29 (-0.3:22.88)<br>n=34	94.36 (40.22)<br>3.49 (-7.59:14.56)<br>n=38	0.1703
**FEV_1/FVC**	54.11 (24.87)<br>0<br>n=91	58.95 (25.79)<br>5.84 (3.52:8.17)<br>n=81	57.02 (27.91)<br>4.76 (2.28:7.24)<br>n=67	63.83 (18.93)<br>5.04 (2.5:7.58)<br>n=63	60.7 (24.58)<br>6.26 (3.87:8.66)<br>n=75	<0.0001
**Post BD FEV_1/FVC**	62.82 (21.32)<br>0<br>n=60	71.82 (9.8)<br>2.86 (0.71:5.01)<br>n=41	71.55 (9.69)<br>1.82 (-0.68:4.31)<br>n=27	69.9 (8.16)<br>1.12 (-1.21:3.44)<br>n=36	68.66 (14.24)<br>2.15 (-0.06: 4.36)<br>n=40	0.2822

Table S2: Extent of OCS dose reduction during treatment with benralizumab in patients grouped according to OCS dose at baseline (≤5 mg/day or >5 mg/day)

**Extent of OCS reduction, OCS dose at baseline ≤5 mg/day (n=26)**	**Month 6 (n=25)**	**Month 12 (n=21)**	**Month 24 (n=21)**	**Month 36 (n=21)**
Any reduction	20 (80%)	20 (95.24%)	18 (85.71%)	19 (90.48%)
≥90 %	14 (56%)	18 (85.71%)	16 (76.19%)	18 (85.71%)
≥75 %	15 (60%)	19 (90.48%)	17 (80.95%)	18 (85.71%)
≥50 %	19 (76%)	20 (95.24%)	17 (80.95%)	19 (90.48%)
≥25 %	20 (80%)	20 (95.24%)	18 (85.71%)	19 (90.48%)
No reduction	5 (20%)	1 (4.76%)	3 (14.29%)	2 (9.52%)
Elimination	14 (56%)	18 (85.71%)	16 (76.19%)	18 (85.71%)
**Extent of OCS reduction, OCS dose at baseline >5 mg/day (n=41)**	**Month 6 (n=39)**	**Month 12 (n=26)**	**Month 24 (n=22)**	**Month 36 (n=24)**
Any reduction	32 (82.05%)	21 (80.77%)	21 (95.45%)	23 (95.83%)
≥90 %	25 (64.1%)	16 (61.54%)	18 (81.82%)	20 (83.33%)
≥75 %	27 (69.23%)	19 (73.08%)	20 (90.91%)	21 (87.5%)
≥50 %	31 (79.49%)	21 (80.77%)	21 (95.45%)	23 (95.83%)
≥25 %	31 (79.49%)	21 (80.77%)	21 (95.45%)	23 (95.83%)
No reduction	7 (17.95%)	5 (19.23%)	1 (4.55%)	1 (4.17%)
Elimination	25 (64.1%)	16 (61.54%)	17 (77.27%)	20 (83.33%)

Figure S1: Number and percentage of patients who achieved and did not achieve CR (either pCR or cCR) at 12, 24, and 36 months during the treatment with benralizumab (values calculated from baseline).

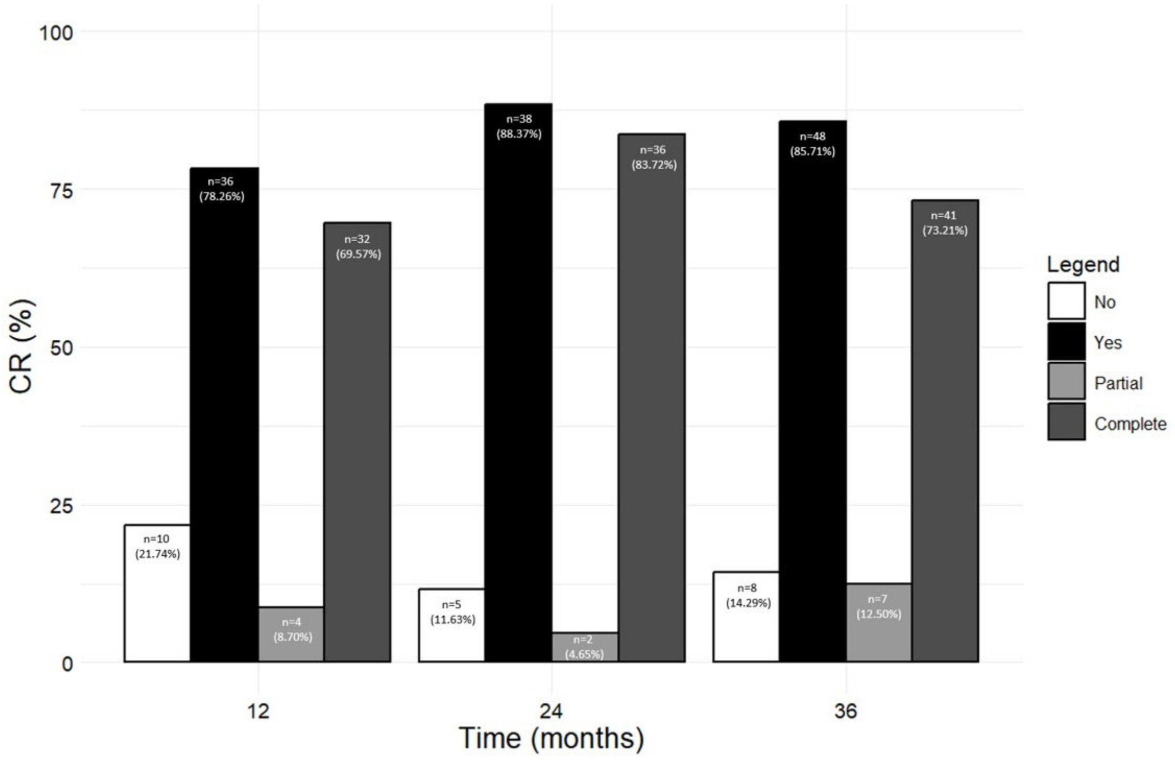

Supplement: Supplementary file 1 [file jcm-13-03013-s001.zip › jcm-2905365-supplementary.pdf]
